# Measuring the Impact of Social Media on Young People's Mental Health: Development and Validation of the Social Media-Induced Tendency Scale

**DOI:** 10.1155/2023/8677521

**Published:** 2023-04-19

**Authors:** Lawrence Ejike Ugwu, Erhabor Sunday Idemudia, Olive O. Chukwu, Maria Chidi Christiana Onyedibe

**Affiliations:** ^1^Faculty of Humanities, North-West University, South Africa; ^2^Psychology Department, Renaissance University, Ugbawka, Enugu, Nigeria; ^3^Psychology Department, University of Nigeria Nsukka, Nigeria; ^4^Physical Activity, Prevention and Cancer, German Cancer Research Center (DKFZ), Im Neuenheimer Field 280, 69120 Heidelberg, Germany

## Abstract

Social media use has been linked to adverse health outcomes such as depression. To facilitate interventions, understanding the varied causes of depression is necessary. The authors developed a social media-induced depression tendency (SMIDT) scale for use with young people and aimed to validate it for young people in Nigeria. The study was conducted in three parts using an online survey (Google Forms) with purposive sampling targeting young people. Study 1 was an exploratory study that developed the SMIDT scale with 361 young people aged 16 to 26 years (mean age = 22.81). A concise measure of SMIDT was obtained. In study 2, confirmatory factor analysis was performed on the SMIDT with young people aged 17 to 25 years (mean age = 23.61). Construct, discriminant, and concurrent validities were established, and three factors were identified (sensitivity/attention seeking, worthlessness, and escapism/reality avoidance), which explained 55.87% of the variance. Study 3 tested the predictive validity of the scale. The results showed that the 15-item SMIDT scale had high internal consistency and satisfactory validity. The SMIDT scale can enable the assessment of factors associated with social media-induced depression tendency. The three factors identified in the scale provide insight into the factors contributing to depression associated with social media use. The SMIDT scale has the potential to help identify at-risk individuals and in-developing interventions to prevent or reduce social media-induced depression tendencies. However, this study only focused on young people in Nigeria. Additional studies using the SMIDT scale are required to assess its generalizability and applicability in evaluating other factors, such as quality of life among young people. Moreover, while social media use has been associated with adverse health outcomes, it is crucial to recognize that it can also positively affect mental health. Further research is necessary to explore the complex relationships between social media use and mental health outcomes.

## 1. Introduction

Social media platforms such as Facebook, Instagram, Snapchat, and TikTok are extremely popular among young people [1] and are the most popular ways to communicate with friends and family for many reasons. It is convenient and easy to reach many friends and family members simultaneously, regardless of location or distance [2]. It is also free to use, which makes it accessible to a wide range of people [3]. It has become common to check social media regularly [4].

Young people's excessive use of social media networks may have reduced face-to-face or real-life social interactions [5, 6]. Additionally, regular exposure to idealized and unrealistic depictions of emotions, lies, and connections on social media can make individuals feel inferior and isolated compared to their peers [7]. This is because they believe that others live happier and more connected lives, making them feel socially disadvantaged.

Young people's use of social media has raised concerns about its impact on their mental health, particularly their life satisfaction and depressive symptoms [8, 9]. Earlier research (e.g., [10], [11]) proposed active-passive social media use, indicating that active (sending messages privately or broadcasting) was more associated with well-being, while passive (browsing other people's posts and profiles) was more associated with ill-being. However, a scoping review by Valkenburg et al. [12] showed that such dichotomy was difficult to establish as both can elicit positive and negative effects.

Depression significantly impacts health and can increase morbidity and mortality [13, 14]. The health effects of this mental disorder are becoming more pronounced, with the World Health Organization [15] reporting that depression is one of the major contributors to disability-adjusted life years globally. This is partly because depression often affects people in their prime years of early adulthood [15].

The cause of depression includes complex interaction between social, psychological, and biological factors [15]. The world is evolving in how we interact and seek connection and support from each other through social media. Unfortunately, this has also created opportunities for misinformation and high expectations from themselves that can be overstated, leaving young people feeling isolated and aiming for unattainable goals [5, 16], which could result in the term social media-induced depression tendency (SMIDT).

SMIDT is the tendency to experience depression due to problematic social media usage. This high-frequency usage has been linked to adverse health-related outcomes, for example, depression, anxiety, and suicide (e.g., [17]). The increasing reduction in in-person social interaction has reduced emotional closeness among young people. The use of social media gives a faulty reflection of reality, where people could be lonely and feel miserable yet portray healthy and lively outlooks [18]. The constant display of picture-perfect lifestyles seen on social media platforms like Facebook and Instagram has been shown to be toxic for young people as they struggle with body image, eating disorders, anxiety, and depression [19].

The increasing negative influence of social media on people's lives, especially depressive symptoms [20, 21], has necessitated this investigation. People tend to be active or passive in social comparison in social media use. This is where people compare themselves (how they look and perceive “better lives” of those they see on social media themselves) and envy and depressive tendencies (wishing to possess the material and status of others) [12, 21, 22].

Unhealthy Internet use has been found to elicit symptoms of depressive episodes like sadness, lack of interest in activities previously enjoyed, and lack of energy, self-confidence, self-blame, suicidal ideation, indecision, and inattention [23]. In addition, studies (e.g., [24]) have found that pathological Internet use was positively correlated with depression.

In most studies of depression, instruments were designed to cut across the lifespan (e.g., Beck Depression Inventory; [25]); children and adolescents (e.g., Behaviour Assessment System for Children; [26]), general adults' population (e.g., Beck Hopelessness Scale; [27]), and older adults (e.g., Geriatric Depression Scale; [28]) have been used. Since specific mental health concerns like postpartum depression and premenstrual dysphoric disorder depression pay attention to circumstantial events in people's lives, there is a need to consider the role of social media in inducing depression in people. However, currently, no instrument exists for measuring social media-induced depression tendencies within the mental health framework to the best of our knowledge. Nevertheless, from the review of previous studies on social media and depression, the following attributes were observed as markers of the link between depression and social media use:

- The problematic use of smartphones increased attention seeking and heightened sensitivity (e.g., [29, 30])
- When little or absence of peer endorsement on social media (in the form of “likes,” “following,” etc.) induces a sense of worthlessness (e.g., [31–33])
- The measurement of achievement by those they see online consequently experiencing depressive tendencies avoiding reality (e.g., [22, 34])

A major limitation of the studies listed above was that they all used Beck Depression Inventory to measure depression related to social media use. Also, they referred to the depression experienced by social media users as depressive tendencies (e.g., [22]). This inference could also mean that the depressive tendency of the individuals might be responsible for their unhealthy social media use. This casts a cloud on the efficiency of the measurement of depression. The present study is aimed at delving into the nature of depression as caused by social media usage as it induces depression in the population.

There is a gap in the current research on social media-induced depression tendencies (SMIDT) among young people. To address this, we created an instrument to measure SMIDT to advance future studies with broader applicability. Understanding the mechanisms behind SMIDT is important to develop effective prevention strategies and interventions. Young people are particularly vulnerable to the negative effects of social media due to their developing sense of self and greater susceptibility to peer pressure and unmonitored exposure [35]. The impact of social media on this group is crucial in addressing their mental health needs. Social media can alter how people interact, and understanding its effect on mental health is crucial for optimizing social interactions [36]. This new scale can be used for self-awareness screening and evaluating the impact of interventions in promoting mental health literacy among young people.

### 1.1. Preliminary Stage: Item Generation

A focus group of undergraduates of different years of study (five students in each class) were asked to give insights on their understanding and the influence of social media on their mental health. From the discussion, some items were generated. Also, a literature review was extensively carried out to add to the item pool. During the focus group discussions (which happened twice on two different days) and literature review, three major domains stood out (sensitivity/attention seeking, worthlessness, and escapism/reality avoidance). The sensitivity/attention seeking factor described the view of expectations of “like,” “view,” and “comments” from their social media activities. The worthlessness factor describes their self-esteem or self-worth, inadequacy, dissatisfaction, and isolation. Escapism/reality avoidance comprises items investigating the need for social compensation and escaping daily boredom and pressure.

Fifty-one items were generated from the focus group discussion and the literature review. The 51 items were further refined and formulated in the form of statements. The three domains that made up the 51 items generated were sensitivity/attention seeking (11 items), worthlessness (20 items), and escapism/reality avoidance (10 items). All items were positively worded. The response option was on a 5-point scale, with 1 = *never*, 2 = *rarely*, 3 = *sometimes*, 4 = *often*, and 5 = *always*.

### 1.2. Content Validity

A panel of six experts was formed to assess content validity, including two social psychologists, two developmental psychologists, and two clinical psychologists. The measurement goal, target population, clear framework definition, and item selection were presented to the panel. The panel also calculated the content validity index (CVI) using a 4-point Likert scale (1 = not relevant, 2 = somewhat relevant, 3 = quite relevant, and 4 = highly relevant) to eliminate a neutral midpoint [37].

The CVI (content validity index) is calculated by dividing the number of experts who rated 3 or 4 by the total number of experts. After two meetings, the original 51 items were evaluated, and any irrelevant or repetitive questions were removed, resulting in a 41-item questionnaire. According to Polit and Beck [38], content validity is based on subjective or professional judgment and must include at least six experts. The I-CVI must be at least .83 for a sample of six experts.

### 1.3. Instrument Development

The SMIDT scale was developed by employing a standardized multistep method [39]. We validated the scale by using a two-stage test (two studies). In study 1, the goal of the data reduction was to develop a comprehensive scale, by conducting an exploratory factor analysis (EFA). In study 2, we examined the final SMIDT scale by conducting a confirmatory factor analysis (CFA), measure for internal consistency, construct validity, discriminant validity, and concurrent validity. In the third study, we tested the predictive validity of the scale.

## 2. Study 1

### 2.1. Method

#### 2.1.1. Participants

We engaged 361 undergraduates who answered the 41-item preliminary questionnaire. They include 193 females (53.46%) and 168 males (46.54%) with a mean age of 22.81 (SD = 4.66) and age range of 16-26 years, who were purposively selected from five universities in the southeast region of Nigeria to test the psychometric properties of the SMIDT scale. Informed consent was obtained from the students before they filled out the questionnaire.

#### 2.1.2. Procedure

Before the study was conducted, the research ethics committee (REC) reviewed and approved the study protocol, informed consent, and questionnaire. The study was approved by the Renaissance University Research Ethics Committee (Reference 10/RNU03/22). Ethical approval is a crucial step in the research process, as it helps to ensure that the study is conducted ethically and responsibly and that the rights and welfare of study participants are protected. It also enhances the credibility and validity of the study findings, indicating that the study was conducted by established ethical standards.

Questionnaires were administered web-based survey (Google Forms). Before any participant could respond to the questionnaire, the participants were asked three eligibility screening questions. Those who responded that they were below 14 years of age, did not reside in Nigeria, and did not attend any university were not eligible to participate and received a thank you message. Those who met the criteria were taken to the next page, where they read the consent form explaining the study's purpose and method and participants' rights. Participation was anonymous, confidential, and voluntary. Without completing the questionnaire, they would not be able to submit it. Due to the nature of the questionnaire that was designed, only fully completed questionnaires were submitted.

#### 2.1.3. Design/Statistical Analyses

The study design adopted was a cross-sectional design. Data were analyzed using SPSS for Windows (version 25.0) for descriptive statistics and exploratory factor analysis (EFA). EFA is a statistical method used to identify underlying relationships among variables. It is used to extract a smaller number of latent variables (factors) that explain most of the variability in the data. These factors can then be used to describe the relationships between the original variables more straightforwardly and interpretably.

#### 2.1.4. Results

**Figure 1:**
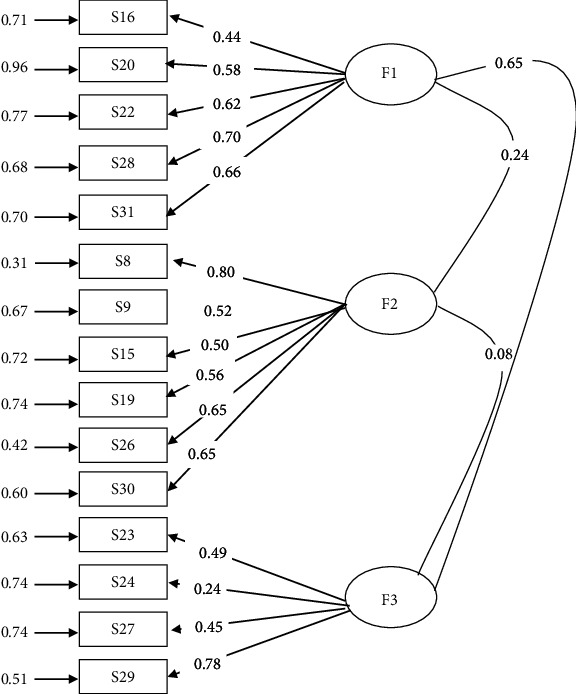
Social media-induced depression tendency model. Confirmatory factor analysis based on 15 items and three factors. Note. F1 = sensitivity/attention seeking; F2 = worthlessness; F3 = escapism/reality avoidance.

Table 1 shows the exploratory factor analysis, which examined a 41-item questionnaire that enabled the reduction of the items to 15 (Table 1 and Figure 1), excluding 26 items for either cross loading or loading below .4. It resulted in a 15-item questionnaire, three common factors with eigenvalues greater than 1.0. The Kaiser-Meyer-Olkin measure of sampling adequacy was .82, which explained 55.87% of the total variance.

## 3. Study 2: Reliability and Validity of the SMIDT Scale

Study 2 revealed whether the final SMIDT scale correctly identified factors that are important for SMIDT of young students. A 15-item SMIDT scale was tested in study 2. The reliability and validity of the questionnaire were confirmed with confirmatory factor analysis (CFA) and concurrent validity [40]. The concurrent validity was done with the depression dimension of the Depression Anxiety Stress Scale (DASS-21) and Big Five Personality Inventory (BFI-10). Personality traits are believed to be related to depression (e.g. [41, 42]), as much as previous studies have linked social media to depression; in this study, SMIDT should be related to personality traits.

### 3.1. Participants

In study 2, 591 participants were recruited with mean age of 23.61 (SD = 4.74) between 17 and 32 years. There were 264 (44.7%) females and 327 (55.3%) males, from 15 tertiary institutions in Nigeria.

### 3.2. Instrument

The short version of the Depression Anxiety Stress Scale-21 (DASS-21) [43] is a self-report measure of anxiety, depression, and stress signals. The depression dimension with 7 items was used. The response options are on a 4-point Likert scale, ranging from 0 = “I strongly disagree” to 3 = “I totally agree.” Lee and Kim [44] reported a reliability coefficient of .90 among young Korean university students. The internal consistency for this study is .81.

### 3.3. Big Five Personality

The Big Five Inventory (BFI-10) is a shortened version of the well-established BFI, consisting of 10 items and allowing for assessment of the five dimensions of personality (openness, conscientiousness, extraversion, agreeableness, and neuroticism), with two items per dimension [45]. Participants rate each item on a 5-point scale, ranging from “strongly disagree” (1) to “strongly agree” (5). The higher the score, the more pronounced the relevant personality dimension becomes, for instance, items such as “is reserved” and “is generally trusting.” The 5-point scale ranges from 1 (strongly disagree) to 5 (strongly agree), with items 1, 7, 3, 4, and 6 reverse scored. Ogunsemi et al. [46] reported an acceptable reliability index of between .79 and .88 for the BFI-10 dimensions and a positive concurrent validity coefficient with the subsections of the Mini International Personality Item Pool Scale. The internal consistency coefficient in this study was .81.

### 3.4. Procedure

The questionnaire was an online Google Forms. Large samples of students were accessed using the students' union bodies. A compulsory field for participants to fill their institutions was added to control for the earlier used five institutions. Just like the first study, a consent form was presented before proceeding with the study questions. Participation was anonymous and voluntary.

### 3.5. Design/Statistical Analysis

In study 2, SPSS 25 was utilized to evaluate the descriptive and concurrent validities of the SMIDT scale and depression, while IBM SPSS AMOS 24 was employed to assess the confirmatory factor analysis (CFA) and goodness-of-fit statistics. SmartPLS 3.3 was used to examine the construct and discriminant validities.

### 3.6. Result

The CFA of the SMIDT scale assessed the relevance of the questionnaire constructed from the outcome of the EFA. A maximum likelihood estimation was employed to evaluate the model to the covariance matrix of the confirmatory dataset. To assess model fit, we utilized the *x*^2^ value and the SRMR (standardized root mean square residual) following Browne and Cudeck's [47] recommendations. The RMSEA (root mean square error of approximation) was also calculated. To further assess model fit, we used the CFI (comparative fit index), GFI (goodness-of-fit index), and NFI (normed fit index).

Generally, the criterion for establishing model fit suggests that CFI, GFI, and NFI values close to .90 represent an acceptable fit and values of .90 or higher indicate a good fit [48].

**Figure 2:**
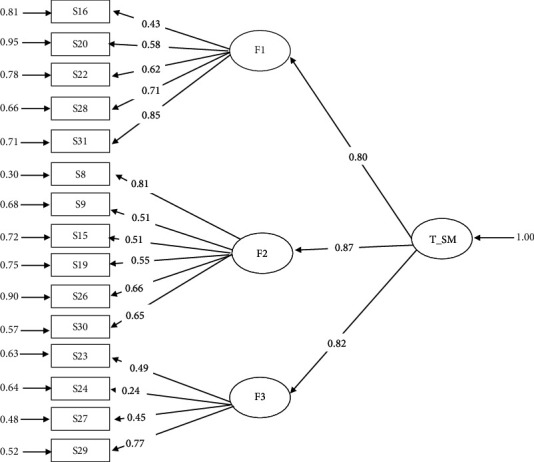
Hierarchical model and factor loadings resulting from confirmatory factor analysis. Note. F1 = sensitivity/attention seeking; F2 = worthlessness/inferiority/negative affect; F3 = escapism/reality avoidance.

CFA was applied to the 15 items identified in the EFA, comparing various models. The correlated three factors produced much better goodness-of-fit statistics (Table 2, Figure 2). To further ascertain a clearer understanding of the individual parameters, additional construct validity analyses were conducted to obtain the item quality (*λ*), Cronbach alpha (CA), composite reliability (CR), and average variance extracted (AVE) presented in Table 3 (part a).

Table 3 (part a) shows the statistical significance of each observation variable with respect to its latent variable factor loading (*λ*). All the factors had a value of .50 or greater, implying that the observed variable accurately reflects its underlying construct. According to Raine-Eudy [49], a CA and CR of .63 are considered good, and all factors in this study exceeded that value. Furthermore, the AVE (average variance extracted) was above the recommended .50 threshold [50], while Table 3 (part b) presents the construct validity for personality dimensions and depression. This showed that they represented satisfactory validity for use in the study.

Table 4 presents estimates for Pearson's correlation, measuring the concurrent validity of the dimensions of SMIDT, the total, personality traits, and depression, to ensure that reflective constructs have stronger relationship with its own indicators than any other construct.

A discriminant validity evaluation was conducted using the heterotrait-monotrait (HTMT) ratio of correlation, where values should not surpass .85, according to Henseler et al. [51].  Table 5 shows that there was no multicollinearity found, and the domains are distinct to each other.

### 3.7. Reported Social Media-Induced Depression Tendency

As shown in Table 6, the overall mean score of the SMIDT scale was 41.64 (SD = 8.44). Analyzing from the 4 dimensions, it showed that overall SMIDT for *sensitivity/attention seeking* is mean ± SD = 13.05 ± 4.02, *worthlessness* is mean ± SD = 18.14 ± 3.36, and *escapism/reality avoidance* is mean ± SD = 10.54 ± 3.23. Finally, the total score on SMIDT difference between males and females was not significant (*t* = −1.63, *p* = .10), same with sensitivity/attention seeking (*t* = 1.01, *p* = .31) except worthlessness (*t* = −3.31, *p* < .001) and escapism/reality avoidance (*t* = −2.19, *p* = .03), where females scored higher compared to the males.

The study found that sensitivity/attention seeking, worthlessness, escapism/reality avoidance, and the composite of the whole scale were all related to certain personality traits and depression. Sensitivity/attention seeking was positively associated with extraversion and negatively related to agreeableness. Conscientiousness and neuroticism were positively associated with sensitivity/attention seeking. Sensitivity/attention seeking was not related to openness. Sensitivity/attention seeking was positively associated with depression. Worthlessness was negatively related to agreeableness and positively associated with neuroticism and openness. Worthlessness was positively associated with depression. Escapism/reality avoidance was positively related to extraversion and neuroticism and negatively related.

## 4. Study 3: Predictive Validity of the SMIDT Scale

### 4.1. Participants

The sample of the third study was used for predictive validity. Predictive validity refers to the ability of a measurement or test to predict a certain outcome or behaviour in the future. It measures how well a test or model can predict the outcome it is designed to measure. In study 3, 658 participants were recruited with a mean age of 21.95 (SD = 3.79) between 16 and 25 years. There were 460 (69.9%) female and 198 (30.1%) male students from 4 tertiary institutions in Nigeria.

### 4.2. Instruments

#### 4.2.1. Social Comparison Order

The participants took part in an 11-item social orientation scale [52] which had two dimensions: comparison of abilities (items 1-6, e.g., “How am I doing?”) and comparison of opinion (items 7-11, e.g., “What shall I feel/think?”). Items 5 and 11 were reverse scored. The items were rated on a 5-point Likert scale from 1 (strongly disagree) to 5 (strongly agree). The internal reliability of the scale was high, with a Cronbach alpha of .72 for comparison of abilities and .71 for comparison of opinion.

#### 4.2.2. State Self-Esteem Scale (SSES)

The State Self-Esteem Scale (SSES) was modified by Heatherton and Polivy [53] from the Janis-Field Feeling of Inadequacy Scale [54]. The SSES is a 20-item scale used to measure temporary changes in self-esteem, consisting of 3 self-esteem factors: performance, social, and appearance. The response options range from 1 (not at all) to 5 (extremely) on a Likert-type scale, with 13 items being reverse scored. High scores indicate high-state self-esteem, while low scores indicate low-state self-esteem. Brito et al. [55] conducted convergent and discriminant validities of the SSES and found it to show a strong, positive, and significant correlation with the Rosenberg self-esteem scale. The internal reliability for performance is .77, social is .72, and appearance is .83.

#### 4.2.3. Social Media Addiction

The Bergen Social Media Addiction Scale (BSMAS) was designed by Andreassen et al. [56] to measure problematic social media use behaviours over 12 months. It consists of 6 items that are rated on a 5-point Likert scale, with options ranging from “very rarely” (1) to “very often” (5). For example, one item asks, “How often have you used social media in the past year to escape personal problems?” The possible total score ranges from 6 to 30, with higher scores indicating a more problematic use of social media. Stănculescu [57] reported a Cronbach's alpha of .84 and acceptable convergent and concurrent validities with the self-esteem. The scale's internal reliability was excellent in the study, with a Cronbach's alpha coefficient of .74.

#### 4.2.4. Emotional Intelligence

The Brief Emotional Intelligence Scale (BEIS-10) measures emotional intelligence and has 10 items. The scale consists of five dimensions: (1) appraisal of own emotions, (2) appraisal of others' emotions, (3) regulation of own emotions, (4) regulation of others' emotions, and (5) utilization of emotions. Participants responded on a 5-point scale, ranging from 1 (strongly agree) to 5 (strongly disagree). Davies et al. [58] reported initial psychometric support for this measure. The responses were recoded so that higher scores indicate higher levels of emotional intelligence. The total score is .77, with scores of .60, .38, .40, .15, and .46 for the five dimensions.

### 4.3. Procedure

The researcher created a Google Forms and shared the link to 4 schools' community social media pages, with the permission of each school's student affair office. All participants were informed that their participation was voluntary and that data would remain confidential; they gave their consent before filling out the form. The form can be filled out within 10 minutes. Six hundred and fifty-eight responded to the online questionnaire.

### 4.4. Design

The design of the study is a cross-sectional survey design. The data for the analysis were analyzed using hierarchical multiple regression. The data obtained from participants were analyzed using the statistical package for the social sciences (SPSS 25.0) shown in Table 7.

## 5. Result

The study found that the SMIDT dimensions were related to social comparison order, State Self-Esteem Scale, social media addiction, and emotional intelligence. Age was negatively correlated with worthlessness and escapism/reality avoidance (ERA). Women were more associated with worthlessness and ERA. Social comparison (ability) was positively correlated with all three dimensions of SMIDT. Social comparison (opinion) was positively correlated with only worthlessness. Social media addiction (SMA) was positively related to all the dimensions of SMIDT. Emotional intelligence (appraisal of own emotions) did not correlate significantly with any of the dimensions of SMIDT. Emotional intelligence (appraisal of others' emotions), regulation of others' emotions, and regulation of own emotions were negatively correlated with all three dimensions of SMIDT. Emotional intelligence (utilization) was positively associated with sensitivity/attention seeking and escapism/reality avoidance but not with worthlessness.

## 6. Discussion

This study is aimed at developing and validating a new instrument for assessing depression among young people in Nigeria. The instrument developed was the SMIDT scale, which is a 15-item questionnaire that measures depression across three factors: sensitivity/attention seeking, worthlessness, and escapism/reality avoidance.

The researchers used two statistical techniques to validate the SMIDT scale: exploratory factor analysis (EFA) and confirmatory factor analysis (CFA). EFA was used to identify the underlying factor structure of the SMIDT scale, while CFA was used to confirm the factor structure in independent samples.

The study involved three samples of young people in Nigeria. Sample 1 comprised 361 participants, sample 2 comprised 591 participants, and sample 3 comprised 658 participants. The three-factor structure identified in sample 1 was subsequently confirmed in samples 2 and 3, indicating that the SMIDT scale has good cross-validation.

The study also demonstrated that the SMIDT scale has good convergent, discriminant, and predictive validities. Convergent validity means that the SMIDT scale is positively correlated with other measures of depression, such as the DASS-21 and the big five personality. Discriminant validity means that the SMIDT scale is not significantly correlated with measures of unrelated constructs, such as anxiety. Predictive validity using the social comparison order, state self-esteem, social media addiction, and emotional intelligence scales means that the SMIDT scale can predict future depression outcomes. Finally, the study showed that the SMIDT scale has good internal reliability and validity across the three samples. Overall, the findings of this study suggest that the SMIDT scale is a reliable and valid instrument for assessing social media-induced depression tendencies among young people in Nigeria.

### 6.1. Practical Implication

This raises concern about the manner social media use influences the mental health of young people. The inculcation of social media literacy and creating awareness to protect mental health safety usage habits among young people in schools. As much as civic education is taught, young people can learn to be empathetic in framing their posts, comments, and reactions.

Social interaction is an art that has been neglected over the years, even as social media technologies have been embraced without considering their adverse effect on young people. Social media platforms are not the safest media to gain social support in increasingly socially isolated societies. Reckless usage can trigger unpleasant feelings that consequently affect the individual's well-being. Also, more restrictions can be built into current technologies to censor languages and visual and audio materials young people consume. It is crucial for individuals, particularly parents and educators, to be aware of the potential adverse effects of social media use and to educate themselves on how to mitigate the risks.

There is a need for effective interventions to address the adverse effects of social media use on mental health. Understanding the underlying mechanisms of social media-induced depression is essential for developing effective interventions. This understanding can help to identify high-risk individuals and develop targeted interventions. The study of social media-induced depression can inform public policy decisions regarding social media use and mental health.

### 6.2. Implications of the Study

This study lays the foundation for further research on the SMIDT scale, which requires additional psychometric evaluation to enhance its comprehensiveness. The validation of the SMIDT instrument requires longitudinal studies to examine the causal relationships between the SMIDT factors and factors such as quality of life, academic performance, suicidal ideation, and self-esteem.

The findings of this study suggest that the SMIDT scale is a suitable tool for measuring social media-induced depression among young people and provide evidence for the factor structure, reliability, and validity of the measures.

### 6.3. Limitations

The current study has some limitations that should be acknowledged. The use of self-reported measures has the potential for bias. Future research should investigate whether self-reported social media-induced depression using the SMIDTs is consistent with other assessment methods, such as clinical interviews. Another limitation is that information was not collected on the specific social media platform used by participants or their socioeconomic status. Further research is needed to explore how these factors may impact responses. Additionally, the SMIDT was primarily developed using young Africans as the sample, and its validity should be tested among older adults and more diverse populations. Lastly, the study's cross-sectional design means that causality cannot be inferred from the results.

## 7. Conclusions

The SMIDT scale is a self-report questionnaire developed to measure depression tendencies due to social media use among young people. The scale consists of 15 items, taking approximately 10 minutes to complete. The study that established the validity of the SMIDT scale found that it is a reliable tool for assessing depression related to social media use among young people.

However, the study has some limitations that may affect the generalizability of its findings. For example, the study only included participants within a narrow age range, which may limit the applicability of the results to older individuals. Additionally, the study did not provide information on the specific types of social media platforms used by the participants, or their socioeconomic status, which may impact their relationship with social media.

To improve the consistency of future studies, the study's authors recommend expanding the age range of participants to include older adults and a more diverse demographic sample. Collecting information on the types of social media platforms used and socioeconomic status may also be beneficial in improving the accuracy of the results. By addressing these limitations, future studies using the SMIDT scale can provide a more comprehensive understanding of the relationship between social media use and depression.

## Figures and Tables

**Table 1:** Exploratory factor analyses of 15-item social media-induced depression tendency scale.

		Component		
		1	2	4
Factor 1	*Sensitivity/attention seeking*			
Sm31	I expect people to pay attention to whatever I post	.71		
Sm20	I want to be popular, that is why I frequently use the social media platform	.70		
Sm28	What my online friends think of me is important to me	.66		
Sm22	It hurts me when one of my “followers” “unfollow” or “unfriends me”	.62		
Sm16	My posts reflect on my current mood	.51		
Factor 2	*Worthlessness/inferiority/negative affect*			
Sm9	Most of the thing I see on social media makes me feel like hurting myself		.82	
Sm8	Seeing young people of my age displaying financial independence makes me feel inferior		.65	
Sm15	Going through my social media pages makes me feel disappointed in myself		.59	
Sm26	Stories of unfortunate events make me feel sad		.80	
Sm30	People can be inconsiderate of others on the social media platforms		.75	
Sm19	I show empathy for others' sad feelings on social media		.67	
Factor 3	*Escapism/reality avoidance*			
Sm24	Social media is full of lonely and depressed people			.65
Sm29	Social media is an excellent place to hide my weaknesses			.61
Sm23	I feel more comfortable speaking online with an online friend than in person			.59
Sm27	Stories of young people committing suicide make it feel like an easy escape from reality			.57

**Table 2:** Confirmatory factor analysis of the social media-induced depression tendency scale.

Model	*x* ^2^	df	*x* ^2^/df	GFI	NFI	CFI	RMSEA	SRMR
Null	287.36^∗∗^	64	3.49	.78	.79	.81	.101	.10
One factor	292.48^∗∗^	65	4.50	.88	.71	.76	.099	.09
Uncorrelated factors	146.17^∗∗^	84	1.74	.95	.87	.94	.045	.05
Correlated factors	104.19^∗∗^	76	1.37	.96	.91	.97	.032	.04
Hierarchical	113.00^∗∗^	78	1.45	.96	.90	.97	.035	.05

Note: ^∗∗^*p* < .001.

**(a):** 

Items	Loading	CA	CR	AVE
Factor 1		.81	.82	.59
Sm31	.68			
Sm20	.73			
Sm28	.82			
Sm22	.81			
Sm16	.67			
Factor 2		.76	.79	.66
Sm9	.60			
Sm8	.82			
Sm15	.79			
Sm26	.99			
Sm30	.62			
Sm19	.61			
Factor 3		.70	.77	.69
Sm24	.51			
Sm29	.90			
Sm23	.61			
Sm27	.60			

**(b):** 

Variables	Dimensions	CA	CR	AVE
Personality	Extraversion	.76	.82	.59
	Agreeableness	.73	.79	.66
	Conscientiousness	.74	.77	.69
	Neuroticism	.79	.86	.71
	Openness to experience	.88	.91	.86
Depression	Depression	.87	.89	.88

**Table 4:** Correlation of the SMIDT (dimensions), total personality, and depression scale.

		*M*	SD	1	2	3	4	5	6	7	8	9	10
1	Extraversion	4.64	1.86	1									
2	Agreeableness	7.39	1.90	-.02	1								
3	Conscientiousness	7.30	1.96	-.04	.22^∗∗^	1							
4	Neuroticism	5.60	1.95	.04	-.06^∗^	-.18^∗∗^	1						
5	Openness	8.07	1.67	-.03	.10^∗∗^	.07^∗∗^	-.15^∗∗^	1					
6	Depression	14.24	5.71	.11^∗∗^	-.17^∗∗^	-.24^∗∗^	.23^∗∗^	-.116^∗∗^	1				
7	F1	13.04	4.05	.13^∗∗^	-.18^∗∗^	.08^∗∗^	.18^∗∗^	.05	.36^∗∗^	1			
8	F2	18.07	3.27	.04	-.20^∗∗^	-.02	.17^∗∗^	.19^∗∗^	.44^∗∗^	.52^∗∗^	1		
9	F3	10.29	3.22	.07^∗^	-.18^∗∗^	-.11^∗∗^	.17^∗∗^	-.02	.41^∗∗^	.44^∗∗^	.43^∗∗^	1	
10	SMIDT	41.40	8.46	.10^∗∗^	-.23^∗∗^	-.01	.32^∗∗^	.05	.46^∗∗^	.85^∗∗^	.80^∗∗^	.76^∗∗^	1

Note: ^∗^*p* < .05 and ^∗∗^*p* < .001. F1 = sensitivity/attention seeking; F2 = worthlessness; F3 = escapism/reality avoidance.

**Table 5:** Heterotrait-monotrait ratio (HTMT).

F1			
F2	.75		
F3	.63	.66	.23

Note: F1 = sensitivity/attention seeking; F2 = worthlessness; F3 = escapism/reality avoidance.

**Table 6:** Means (SD) SMIDT scores by the gender.

Scales	Total	Male	Female	*t*	*p*
Total	41.65 (8.44)	41.01 (8.68)	42.16 (8.22)	-1.63	.10
F1	13.05 (4.02)	13.24 (4.15)	12.90 (3.91)	1.01	.31
F2	18.14 (3.36)	17.64 (3.16)	18.55 (3.47)	-3.31	<.01
F3	10.45 (3.23)	10.13 (3.16)	10.71 (3.26)	-2.19	.03

Note: F1 = sensitivity/attention seeking; F2 = worthlessness; F3 = escapism/reality avoidance.

**Table 7:** Correlation of the SMIDT (dimensions), social comparison order, State Self-Esteem Scale, social media addiction, and emotional intelligence.

S/N		Variables			1	2	3	4	5	6	7	8	9	10	11	12	13
1		Age	21.95	3.79	1												
2		Gender			.20^∗∗^	1											
3	SC	Ability	17.65	4.68	-.17^∗∗^	-.04	1										
4		Opinion	17.93	2.64	-.03	-.14^∗∗^	.08^∗^	1									
5		SMA	22.41	5.17	-.18^∗∗^	-.08^∗^	.44^∗∗^	.07	1								
6	EI	AP-O-E	4.79	1.95	-.15^∗∗^	-.06	-.04	.06	.02	1							
7		AP-OT-E	7.10	1.84	.09^∗^	.10^∗^	-.26^∗∗^	-.07	-.21^∗∗^	-.04	1						
8		REG O-E	6.72	1.88	.38^∗∗^	.22^∗∗^	-.11^∗∗^	-.05	-.20^∗∗^	-.04	.18^∗∗^	1					
9		REG OT-E	5.92	1.94	-.35^∗∗^	-.28^∗∗^	.23^∗∗^	-.03	.22^∗∗^	.03	-.07	-.39^∗∗^	1				
10		Utilization	7.89	1.63	.25^∗∗^	.17^∗∗^	.04	.05	-.02	-.22^∗∗^	.09^∗^	.27^∗∗^	-.19^∗∗^	1			
11	SMIDT	SAS	13.14	3.87	-.03	.01	.36^∗∗^	.03	.81^∗∗^	-.01	-.48^∗∗^	-.31^∗∗^	.39^∗^	.10^∗∗^	1		
12		Worthlessness	18.43	3.30	-.09^∗^	-.12^∗∗^	.38^∗∗^	.34^∗∗^	.82^∗∗^	.01	-.33^∗∗^	-.42^∗∗^	.37^∗∗^	.02	.59^∗∗^	1	
13		ERA	10.85	3.75	-.30^∗∗^	-.09^∗^	.49^∗∗^	-.00	.72^∗∗^	.03	-.28^∗^	-.53^∗∗^	.25^∗∗^	-.16^∗∗^	.31^∗∗^	.38^∗∗^	1

^∗∗^ *p* < .001 and ^∗^*p* < .05; gender (dummy coded “0,” female; “1,” male). SC: social comparison; EI: emotional intelligence; SMIDT: social media-induced depression tendency; SMA: social media addiction; AP-OE: appraisal of own emotion; AP-OT-E: appraisal of others' emotion; REG-OT-E: regulation of others' emotions; REG-O-E: regulation of own emotions; SAS: sensitivity/attention seeking; ERA: escapism/reality avoidance.

## Data Availability

The datasets generated during and/or analyzed during the current study are available from the corresponding author on reasonable request.
